# Background selection as null hypothesis in population genomics: insights and challenges from *Drosophila* studies

**DOI:** 10.1098/rstb.2016.0471

**Published:** 2017-11-06

**Authors:** Josep M. Comeron

**Affiliations:** 1 Department of Biology, University of Iowa, Iowa City, IA 52242, USA; 2 Interdisciplinary Program in Genetics, University of Iowa, Iowa City, IA 52242, USA

**Keywords:** background selection, selective sweep, genetic hitchhiking, recombination

## Abstract

The consequences of selection at linked sites are multiple and widespread across the genomes of most species. Here, I first review the main concepts behind models of selection and linkage in recombining genomes, present the difficulty in parametrizing these models simply as a reduction in effective population size (*N*_e_) and discuss the predicted impact of recombination rates on levels of diversity across genomes. Arguments are then put forward in favour of using a model of selection and linkage with neutral and deleterious mutations (i.e. the background selection model, BGS) as a sensible null hypothesis for investigating the presence of other forms of selection, such as balancing or positive. I also describe and compare two studies that have generated high-resolution landscapes of the predicted consequences of selection at linked sites in *Drosophila melanogaster*. Both studies show that BGS can explain a very large fraction of the observed variation in diversity across the whole genome, thus supporting its use as null model. Finally, I identify and discuss a number of caveats and challenges in studies of genetic hitchhiking that have been often overlooked, with several of them sharing a potential bias towards overestimating the evidence supporting recent selective sweeps to the detriment of a BGS explanation. One potential source of bias is the analysis of non-equilibrium populations: it is precisely because models of selection and linkage predict variation in *N*_e_ across chromosomes that demographic dynamics are not expected to be equivalent chromosome- or genome-wide. Other challenges include the use of incomplete genome annotations, the assumption of temporally stable recombination landscapes, the presence of genes under balancing selection and the consequences of ignoring non-crossover (gene conversion) recombination events.

This article is part of the themed issue ‘Evolutionary causes and consequences of recombination rate variation in sexual organisms’.

## 1. Introduction

Selection at a given genomic site has evolutionary consequences for genetically linked sites, either neutral or under selection themselves. These consequences of selection at linked sites, however, are multiple and strongly dependent on the selective regime and type of data under study. As such, ‘selection at linked sites’ is not a homogeneous phenomenon and the evolutionary outcomes may overlap less than is often assumed.

Below, I first present the main concepts behind models of selection and linkage with particular focus on the expected consequences for patterns of diversity across recombining genomic regions and how these models may differ in their estimates of the population parameter ‘effective population size’ (*N*_e_). Based on recent genome-wide studies and a re-analysis of a published dataset in *Drosophila melanogaster*, I later propose that the background selection model (BGS; [1–4]) should be considered as a default conceptual framework and its predictions across genomes as a null hypothesis in population genomics studies. Finally, I describe several limitations, challenges and potential biases in studies of selection and linkage. Throughout, I will use the term ‘selection at linked sites’ rather than ‘linked selection’ to emphasize that (i) linkage refers to a genetic property between genomic sites that do not recombine freely during meiosis (as opposed to an association between selective events) and (ii) the consequences of selection at a site can alter population dynamics at both neutral and non-neutral sites.

Fisher [5] and Muller [6] discussed one of the first proposed consequences of selection at genetically linked sites in terms of selection at a polymorphic site *interfering* with selection at a second polymorphic site. Hill & Robertson [7] quantified this phenomenon and showed that selection acting on a segregating variant causes a reduction in the probability of fixation of a beneficial mutation at a linked site. This reduction in efficacy of selection at a site due to selection acting at nearby sites is known as the ‘Hill–Robertson effect’ (HRE; [7–9]). Moreover, and because the probability of fixation of a beneficial mutation with selection coefficient *s* decreases when the product *N*_e_ × *s* decreases [10–12], Hill & Robertson viewed their results in terms of a reduction in *N*_e_ relative to single-locus predictions of selection.

**Figure 1.**
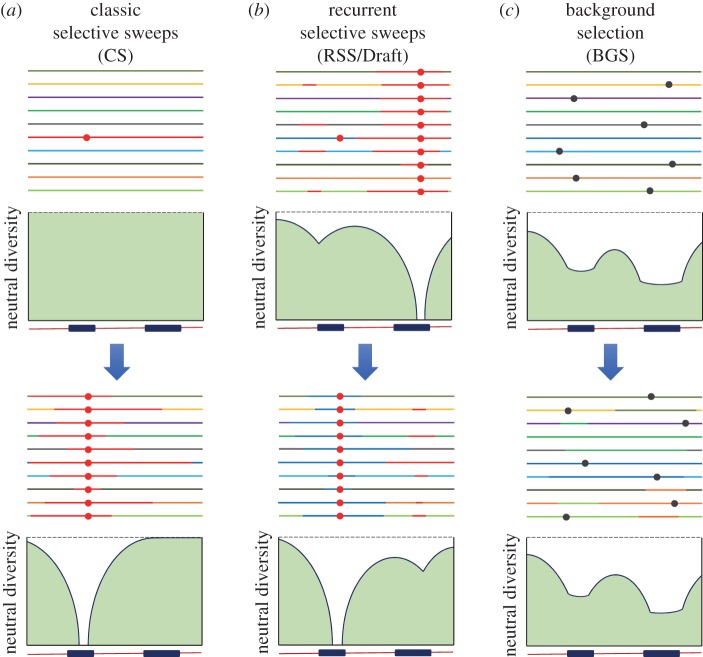
Models of selection with recombination. Horizontal lines represent different genetic backgrounds or haplotypes across a genomic region. Panels below the haplotypes depict qualitative levels of neutral diversity across the region, with the dashed line representing the expected level of neutral diversity in the absence of selection. Blue rectangles below the neutral diversity panels represent the location of two functionally relevant sequences where beneficial and deleterious mutations can occur. (*a*) CS and (*b*) RSS (or Draft) involve the fixation of new beneficial mutations (red circles) together with linked genetic variants. As a consequence, neutral diversity near the genomic location of the beneficial mutation is strongly reduced immediately after fixation and is expected to recover with time. The RSS/Draft model assumes that selective sweeps can occur before the complete recovery of neutral diversity from a previous sweep. (*c*) BGS predicts the continual appearance and elimination of deleterious mutations (black circles, shown here before being eliminated by selection) together with linked genetic variants. The deleterious mutation rate at functional sequences is assumed to be much higher than the beneficial mutation rate.

Another category of models focuses on how selection changes levels of neutral diversity at linked sites; these are the so-called genetic hitchhiking models. In 1974, Maynard Smith & Haigh [13] described the dynamics of a beneficial variant increasing in frequency together with a variant at a linked site. This process eliminates segregating variation (diversity) near the site of the beneficial mutation once it reaches fixation (the classic selective sweep (CS) model). The size of the genomic region showing a reduction in diversity depends on the strength of selection (increasing size when *s* increases) and the recombination rate (decreasing size when the rate of meiotic recombination per base pair increases) [13,14]. In recombining chromosomes, therefore, genetic diversity is predicted to increase with genetic distance from the position of the recently fixed beneficial mutation, producing a ‘valley’ of diversity characteristic of the CS model (figure 1). Because beneficial mutations are assumed to be rare, the CS model predicts a very dynamic process with highly variable levels of diversity over time and across genomes. The CS model was later expanded by Wiehe & Stephan [15] and Gillespie [16] to include a constant input of beneficial mutations and recurrent selective sweeps (RSS or ‘Draft’ models). Although the target of selection may vary along a chromosome, Draft models forecast a more steady reduction in diversity due to genetic hitchhiking than CS models when analysing large genomic regions (figure 1). Charlesworth and co-workers [1,3,4] proposed a similar phenomenon describing the consequences of selection eliminating linked neutral diversity due to genetic hitchhiking, but with the critical difference that the cause of hitchhiking is the continuous removal of deleterious mutations (BGS; see also Hudson & Kaplan [2,17]).

Because neutral diversity in finite populations is an increasing function of *N*_e_ × *u* (where *u* is the mutation rate/bp/generation), the consequences of CS/Draft and BGS models have been also presented in terms of a reduction in *N*_e_ near the sites under selection, in this case relative to predictions in the absence of selection. Not all forms of selection, however, predict a reduction in linked genetic variation and local *N*_e_. Balancing selection, for instance, maintains multiple variants for long evolutionary times and increases diversity at closely linked neutral sites [18–23]. Associative overdominance, in more general terms, can enhance variability at linked sites [24–27]. As such, balancing selection and associative overdominance can be viewed as genetic hitchhiking models that would cause local increases in *N*_e_, more noticeable in genomic regions with reduced recombination [21,25].

Across genomes and chromosomes, all models of selection and linkage predict a variable influence of genetic hitchhiking (and, therefore, variable local *N*_e_) as a result of variable recombination rates. More specifically, CS/Draft and BGS genetic hitchhiking models predict a positive correlation between recombination rates and neutral diversity (*N*_e_ × *u*) across genomes. This qualitative prediction was first confirmed in *Drosophila* [28–33] and has been observed in most other species analysed [34–45]. Moreover, it is now better understood that hitchhiking models predict that local *N*_e_ (ultimately at the resolution of single genomic sites) will vary across genomes not only with recombination rates but also with differences in the distribution of sites under selection (e.g. gene distribution and the intron–exon structure of genes) [35,46–50]. The generality of the positive correlation between diversity and recombination rates also suggests that balancing selection and associative overdominance play secondary roles in explaining the levels of genetic variation across recombining chromosomes.

Quantitative estimates of the magnitude and form of selection causing these general trends across genomes are, however, less straightforward. For, instance, most population genetic models accept that the actual census population size *N* can be (much) greater than the effective population size in the absence of selection (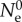) owing to factors such as temporal variation in population size, variance in fecundity and sex ratios. Nevertheless, different models of selection and linkage are differentially sensitive to the disparity between 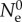 and *N* [51]. More notably, the predicted ‘*N*_e_’ within different models (CS/Draft, BGS or HRE) is not equivalent, given that each model captures different aspects of population dynamics [52–54]. The diversity-related *N*_e_ within CS/Draft models is not mathematically equivalent to an *N*_e_ representing a population with constant size [52,55,56]. Likewise, the *N*_e_ associated with a reduction in diversity within the BGS model is not equivalent to that explaining a reduction in the probability of fixation within HRE models [52,57]. In this regard, it is arguable that an equivalency of *N*_e_ and *N*_e_ × *s* among models has been abused and can generate a number of confusing interpretations when used interchangeably [52–54]. This last point can be particularly problematic when trying to model the consequences of BGS/Draft on diversity using HRE-related estimates of *N*_e_ × *s* from divergence data.

Moreover, as expanded below, inferences about the effects of linkage on rates of evolution of non-neutral mutations need to assume temporal constancy in recombination landscapes, which is not always the case [58–62]. Additionally, demographic events can alter the relative differences in *N*_e_ across the same genome. Combined, it may be safe to point out that estimates of the parameters associated with selection at linked sites can be less direct and less equivalent among models and datasets than is often accepted.

Finally, it is worth noting that HRE and the different genetic hitchhiking models are not mutually exclusive. With very weak selection and tight linkage, HRE will reduce the efficacy of selection removing deleterious mutations and, therefore, BGS models can overestimate the predicted impact reducing diversity [4,52,63,64]. Equivalently, interference between beneficial mutations can limit the rate of adaptation and, therefore, the diversity-reducing effects predicted by Draft models [65–67]. Moreover, in the case of non-recombining genomes, random genetic drift can cause the fixation of weakly deleterious mutations owing to Muller's ratchet [68–73]. Furthermore, under specific non-recombining conditions, Muller's ratchet can explain low levels of diversity and a reduction in the rate of adaptation (see [71,74] and references therein). Whereas all these factors are important for the interpretation of patterns of diversity and adaptation in asexual species as well as in non-recombining genomic regions such as Y chromosomes, below I will focus on the influence of selection on levels of diversity in recombining genomic regions.

## 2. The background selection model as baseline for studying diversity across recombining genomes

Certainly, beneficial mutations are essential in the evolution of species but a much higher number of non-neutral mutations *must be* deleterious [75]. Accordingly, the use of models that incorporate neutral and deleterious mutations as a null alternative to investigate the potential presence of other forms of selection has been a hallmark of evolutionary studies for almost 50 years. Specifically, predictions of the neutral theory of molecular evolution [76], which allows for neutral and strongly deleterious mutations, as well as later models that include weakly deleterious mutations [77,78], have been used as null hypothesis in molecular population genetic analyses, and other forms of selection are accepted when these predictions are incompatible with the data.

It is, therefore, also sensible to use BGS as a conceptual framework and its predictions of diversity across genomes as null hypothesis when testing for alternative selective regimes using population genomics data [3,36,79], now without the assumption of independence between sites. The use of high-resolution BGS predictions of diversity facilitates the identification of outlier genomic regions that show significantly higher or lower diversity than expected, suggesting the action of balancing selection or recent adaptive events, respectively [79]. However, this approach would only be valid if the BGS model could explain a large fraction of the observed variation in diversity across genomes. Recent advances in generating high-resolution recombination maps together with comprehensive genome annotations are now allowing this type of preliminary studies in a number of species [36,79–82].

## 3. How good is the background selection model at explaining the distribution of diversity across genomes? Lessons from *Drosophila melanogaster*

Charlesworth [3] used theoretical predictions of the BGS model to investigate whether naturally occurring deleterious mutations and variation in recombination rates across the genome could account for the observed heterogeneity in levels of nucleotide diversity in *D. melanogaster* (see also Hudson & Kaplan [2]). These initial analyses estimated the magnitude of BGS (estimates of the parameter *B*, 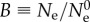) by using relatively crude information on variation in recombination rates and assuming a uniform distribution of deleterious mutations along chromosomes. Despite these approximations, the results showed that BGS is a realistic explanation for the observed reduction in neutral diversity on the fourth achiasmate (non-recombining) chromosome and near centromeres of recombining chromosomes, particularly when the deleterious consequences of transposable element (TE) insertions were taken into account [3].

More recently, Charlesworth [83] estimated the magnitude of BGS in the middle of the X chromosome and autosomes of *D. melanogaster* and *Drosophila pseudoobscura.* By taking into account overall differences in recombination rates between chromosomes and variable selection at exons, introns, untranslated regions (UTRs) and intergenic regions, this study showed that BGS could explain observed differences in diversity levels on the X chromosome relative to autosomes (i.e. the ratio *π*_X_/*π*_A_). In 2014, Comeron [79] further expanded this approach and estimated the predicted effects of BGS models at every genomic position of the *D. melanogaster* genome by combining (i) the actual genomic distribution of all regions putatively under selection (UTRs, exons and introns, overlapping coding sequence (CDS), non-coding RNA (ncRNA) and TEs), (ii) the variable incidence of selection at exons, introns, UTRs, ncRNA, TEs and intergenic regions, (iii) the potential cumulative effect of every position across a chromosome arm when estimating *B* at a site and (iv) different selective and mutational parameters. As in Charlesworth [83], deleterious mutation rates and the distribution of fitness effects (DFEs) were estimated using datasets of diversity that were independent of the ones used to study the potential effects of BGS. Additionally, this study used high-resolution recombination rates [40] and explored the influence of crossover (CO) and non-crossover (NCO, or gene conversion) recombination events on the distribution of BGS effects (see below). This comprehensive approach generated whole-genome high-resolution landscapes of the consequences of selection at linked sites under BGS (i.e. BGS landscapes or *B*-maps) that were then used to evaluate the general fit to the observed levels of neutral diversity and to identify outlier regions [79]. Rank correlation analyses (based on Spearman's *ρ*) between estimates of *B* and observed levels of nucleotide diversity at silent sites (*π*_sil_) suggest that BGS landscapes do a very good job of explaining the observed diversity in *D. melanogaster*. For instance, analyses at the scale of 100 kb show *ρ*^2^ = 0.59 between *π*_sil_ and *B* across autosomes (see [79] for results at different genomic scales).

**Figure 2.**
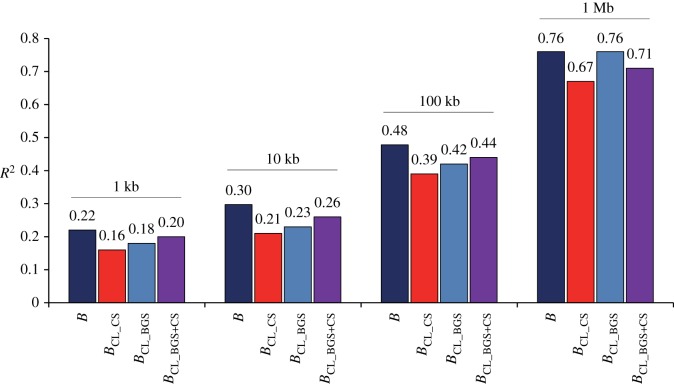
Summaries of goodness of fit between high-resolution estimates of the strength of selection at linked sites across the *D. melanogaster* genome and levels of diversity (*π*) at synonymous sites. *B* indicates estimates of BGS following the methodology presented in [79]. *B*_CL_BGS_, *B*_CL_CS_ and *B*_CL_BGS+CS_ indicate CL-based estimates of *B* from Elyashiv *et al.* [82] when including deleterious mutations, beneficial mutations or the joint effects of deleterious and beneficial mutations, respectively. Estimates of the coefficient of determination (*R*^2^) are shown for analyses of non-overlapping regions of 1, 10, 100 and 1000 kb across autosomes. *R*^2^ estimates for *B*_CL_BGS_, *B*_CL_CS_ and *B*_CL_BGS+CS_ are from Fig. 2 in [82] (see the text for details). In all cases, only regions with recombination rates greater than 0.1 cM per Mb were analysed.

Elyashiv *et al*. [82] have applied an alternative approach to improve the study of selection at linked sites across the *D. melanogaster* genome. Expanding the methodology developed by McVicker *et al*. [36] (see also [84]), these authors inferred selection parameters by maximizing the composite-likelihood (CL) for the observed levels of neutral diversity along the genome. Importantly, Elyashiv *et al*. [82] applied this approach to models with deleterious mutations (a BGS scenario), with beneficial mutations (a CS scenario) or their joint effects (BGS + CS). Together with CL calculations of selective parameters from diversity and divergence data, the authors incorporated properties (i) to (iv) described above and high-resolution genome-wide CO rates to generate detailed landscapes of estimates of *B* (denoted here as *B*_CL_BGS_, *B*_CL_CS_ and *B*_CL_BGS+CS_). Their study also shows that BGS can explain a large fraction of the observed variation in diversity across autosomes. Analyses of 100 kb regions show goodness-of-fit estimates of the coefficient of determination (*R*^2^) between *B*_CL_BGS_ and levels of diversity at synonymous sites (*π*_syn_) of 0.42 (see figure 2 with data from Elyashiv *et al.* [82] for results at different genomic scales). Interestingly, Elyashiv *et al*. show that a model allowing for deleterious and beneficial mutations (BGS + CS) can improve the overall fit even further when the resolution of the study is 100 kb or smaller. At the same time, this study shows that estimates of *B*_CL_CS_ perform consistently worse at explaining diversity levels than either *B*_CL_BGS_ or *B*_CL_BGS+CS_ (figure 2).

A direct comparison of results from the two *D. melanogaster* studies is, however, not necessarily appropriate because estimates of the explained variance from rank correlations such as Spearman's *ρ*^2^ (in [79]) may differ from those from *R*^2^ (in [82]). To have more comparable estimates of fit, I reanalysed the data from Comeron [79] to obtain *R*^2^ between *B* and diversity, focusing on neutral synonymous sites and limiting summaries of goodness of fit to regions with recombination greater than 0.1 cM per Mb, as in [82]. The comparison of analyses of fit shows that *B* landscapes based on BGS models implemented in [79] have a higher or equal explanatory power (higher or equal *R*^2^) describing variation in autosomal diversity than CL approaches based on BGS (*B*_CL_BGS_; [82]) at all physical scales analysed (figure 2).

Combined, the results of these studies in *D. melanogaster* provide three main lessons: (i) all genomic regions, including those with high recombination rates, are likely influenced by BGS, (ii) predictions of BGS show an impressive fit to diversity data at intermediate and large genomic scales, thus supporting the need for considering BGS when evaluating the presence of additional forms of selection, and (iii) when using the same methodological framework, models of BGS + CS are a better explanation for the observed genomic distribution of diversity than models of BGS alone. This latter point is in agreement with previous population genetic studies in this species that detected severely reduced levels of diversity in a number of genomic regions with average recombination rates [85–91] and with the presence of significant outlier regions with lower diversity than expected when using BGS as baseline [79].

In all, BGS, and models of selection and linkage in general, are an active area of research and the approach proposed by Elyashiv *et al*. [82] puts forward a valuable framework for future studies of selection in natural populations. As a guide for these studies of selection at linked sites, I present and discuss below a number of potential limitations, caveats and challenges that should be considered and, ideally, addressed.

### (a) Influence of demographic events

Selection can distort estimates of demographic events. BGS, for instance, generates a consistent excess of low-frequency variants at neutral sites resulting in negative Tajima's *D* [64,92–99]. The predicted magnitude of the skew in the frequency of variants is particularly evident when incorporating DFEs with weakly selected mutations and when recombination is reduced or absent, but it is also expected for a wide range of recombination rates as long as the number of sites under selection is high. In fact, an excess of rare mutations due to BGS is expected across most of the genome for species with a range of recombination rates like *D. melanogaster* [79,100]. These patterns of polymorphism predicted by BGS models and confirmed by simulations could be easily understood as evidence of population expansion and BGS is also likely to bias inferences about most other demographic events [100,101]. At the same time, demography can influence estimates of parameters associated with selection at linked sites [102–104]. In this regard, work by Zeng and co-workers [97,102,105,106] on estimating the joint effects of BGS and demography may help to improve the parametrization of selection coefficients and, ultimately, a default BGS framework.

It is, however, tempting to assume that demographic events should affect different genomic regions similarly and, therefore, play a minor role in inferences of selection once genome-wide patterns are taken into account. This common assumption is not correct when considering that models of selection and linkage predict variable *N*_e_ across genomes, and that the dynamics and consequences of demographic changes depend on population size. The idea that demographic events are not expected to influence different genomic regions similarly follows arguments put forward in studies of neutral diversity on the X chromosome relative to autosomes or in comparisons of mitochondrial relative to autosomal genes when populations undergo demographic changes [107–112]. I argue that the same should be expected across recombining chromosomes as a consequence of variation in *N*_e_ due to genetic hitchhiking.

**Figure 3.**
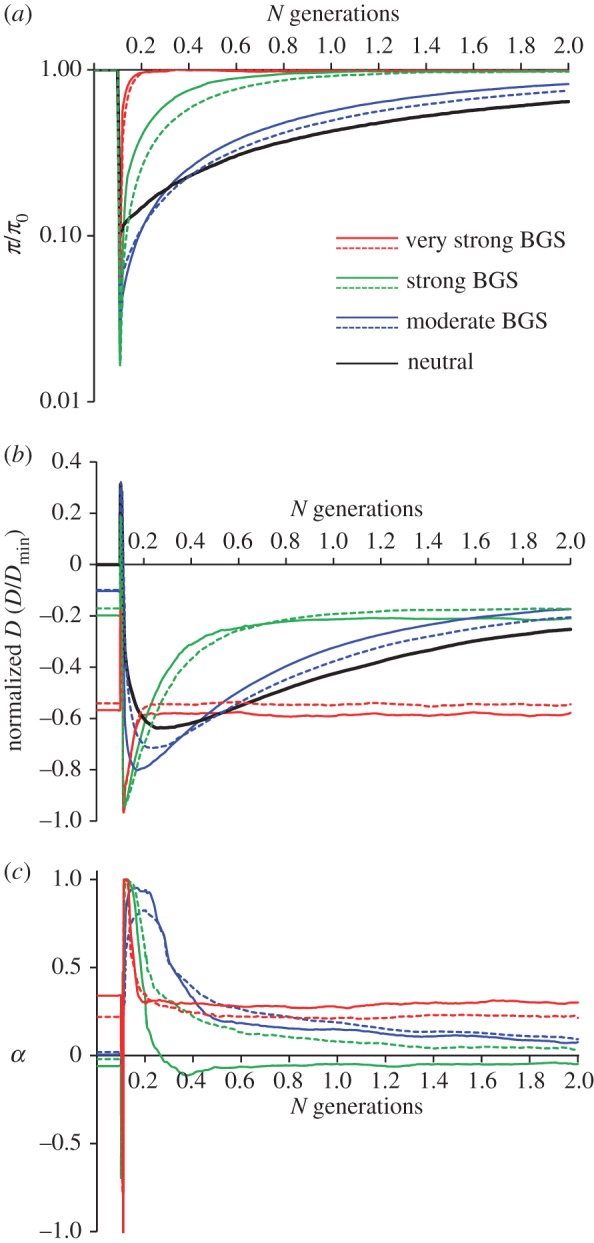
Population dynamics after a bottleneck and rapid recovery under varying intensities of BGS. (*a*) The relative change in diversity at neutral sites (*π*/*π*_0_, where *π*_0_ indicates neutral diversity at equilibrium), (*b*) estimates of Tajima's *D* at neutral sites after normalizing by *D*_min_ (*D*/*D*_min_) [96,113] and (*c*) estimates of *α*, the fraction of adaptive amino acid substitutions [114–117]. All results are based on forward simulations of a panmictic population of 10 000 diploid individuals (*N*) with a severe bottleneck at time 0.1*N* (0.22% of initial population size) and rapid recovery to the initial *N* after 0.01*N* generations. Simulations using the program SLiM [118] followed a chromosome segment of 2 Mb that contains one representative *Drosophila* protein-encoding gene every 10 kb (solid lines) or every 50 kb (dashed lines). Different degrees of BGS were generated through the use of different rates of total recombination observed across *D. melanogaster* chromosomes. Very strong BGS was accomplished with very low rates of CO (*c*; *N* × *c* = 1 × 10^−4^/bp/generation; red line), strong BGS was accomplished with low rates of CO (*N* × *c* = 1 × 10^−3^/bp/generation; green line) and moderate BGS was accomplished with a *D. melanogaster* genome-wide average rate of CO (*N* × *c* = 1.2 × 10^−2^/bp/generation; blue line). All simulations also included an NCO rate (*g*) of *N* × *g* = 4.8 × 10^−2^/bp/generation [40]. Black lines in (*a*) and (*b*) indicate results for neutral sequences not influenced by selection. Estimates of the fraction of adaptive amino acid substitutions *α* were obtained using the DFE-alpha programs [116,117] after jointly inferring the DFEs on amino acid mutations and demography under a two-epoch model. See electronic supplementary material for details on SLiM simulations and analyses.

To exemplify the point presented above, I used forward simulations to explore the consequences of a population going through a severe bottleneck and fast recovery at genomic regions under varying intensities of BGS. Figure 3 depicts the temporal dynamics for (panel *a*) the relative change in diversity at neutral sites, (panel *b*) relative Tajima's *D* at neutral sites (*D*/*D*_min_) [96,113] and (panel *c*) the fraction of adaptive amino acid substitutions (*α*; [114–117]). Different degrees of BGS were generated by using a range of rates of recombination realistic for *D. melanogaster*: very low (but non-zero) recombination (very strong BGS, red line), low recombination (strong BGS, green line) and genome-wide average recombination (moderate BGS, blue line). Moreover, for each of the three recombination levels, lower (dashed lines) and higher (solid lines) degrees of BGS were generated as a consequence of having lower and higher density of sites under selection (figure 3). For any given time point, the different lines should be taken as exemplars of genomic regions across a chromosome under different degrees of BGS (from dashed blue line to continuous red line representing weakest and strongest degrees of BGS, respectively).

At the time of the bottleneck, regions with initially stronger BGS (with more skewed frequency spectrum of neutral variants) will subsample relatively fewer variants and, as a result, analyses shortly after the bottleneck could overestimate the consequences of genetic hitchhiking. More notable is the different speed of approach to equilibrium shown by the different regions [111,112]. Because the time to reach equilibrium after a severe bottleneck mainly depends on the final *N*_e_, regions with stronger local BGS and, therefore, smaller local *N*_e_ will reach equilibrium much faster than those with weaker local BGS and greater *N*_e_. For long periods (in the hundreds of thousands of years for *Drosophila* owing to its large population size), regions with weak BGS will likely exhibit more extreme non-equilibrium patterns than those with moderate or strong BGS. Figure 3*a* shows that regions with high recombination (moderate BGS, blue lines) are much slower in recovering expected levels of neutral diversity than those with low and very low recombination (green and red lines, respectively). Figure 3*b* shows these dynamics for neutral mutations in terms of frequency spectra. For long periods of time, genomic regions with moderate levels of BGS (e.g. blue lines) will show a more negative Tajima's *D* than those with stronger BGS (e.g. green lines). Moreover, neutral mutations in regions with lower density of sites under selection (dashed lines) also tend to show a more negative Tajima's *D* than those in regions with more sites under selection (solid lines) when recombination rates are equivalent. In fact, these results suggest that neutral sites embedded in genomic regions with low BGS (e.g. intergenic regions) may exhibit patterns reminiscent of those caused by selective sweeps when compared with neutral sites under relatively stronger BGS (e.g. synonymous sites or short introns) simply as a consequence of the predicted longer periods of time to equilibrium in the former regions.

These different dynamics also influence estimates of *α* for amino acid substitutions even when using methods that take into account potential changes in population size [116,117,119]. For regions that are considered to have non-reduced recombination rates in *D. melanogaster* (greater than 0.1 cM per Mb; blue and green lines), non-equilibrium creates a tendency for *α* to be positive. Moreover, the weaker the strength of BGS, the longer it takes for *α* to reach the expected equilibrium values (close to zero). Note that for regions with very strong BGS (red lines), the joint estimate of *α* and demography causes positive *α* at equilibrium, whereas estimates of *α* assuming constant population size show equilibrium with *α* ∼ 0. Combined, figure 3 not only shows that demographic events in species with detectable BGS can generate patterns of diversity that vary across the genome, but also that these patterns can be qualitatively different than those predicted at equilibrium.

The simulation study shown in figure 3 represents only one of the many possible demographic events occurring in natural populations, but illustrates the point that qualitative and quantitative interpretations of selection across genomes based on diversity data may be influenced by the joint effects of BGS and demographic events. Moreover, these results emphasize that for long periods of time, every genomic region may be at different stage of its temporal dynamics after demographic events, with some regions showing diversity patterns that can be interpreted as evidence of recent selective sweeps. That is, because every genomic region across recombining chromosomes is probably subject to a different intensity of BGS, it should be analysed separately when studying patterns of demography and selection.

Future models of genetic hitchhiking should, therefore, consider genome-wide as well as region-specific temporal changes in *N*_e_. Complementary studies could also take advantage of advances in optimizing forward simulations that can incorporate BGS and demographic events (e.g. SLiM [118,120,121] and SFS_CODE [122,123]). Machine learning approaches to studying jointly demography, selection and linkage are similarly exciting avenues of research [124–126] and offer new opportunities to better evaluate the causes and consequences of selection at linked sites across genomes.

### (b) Influence of temporal variation in recombination landscapes

Recombination rates and their distribution across genomes vary between species, among populations and among individuals of the same population. Studies by Noor and co-workers [41,59,127] have shown that recombination rate variation within and between *Drosophila* species is more extreme when comparing rates at fine (sub-megabase) genomic scale. This strong dependency on the genomic scale of conservation of recombination rates has now been observed in many species [40,41,58,59,61,62,127–130]. Under models of selection and linkage, temporal changes in recombination rates predict that *N*_e_ at a given genomic region could also change over time, without invoking demographic events. Moreover, this change in *N*_e_ may be associated with a genome-wide change in recombination or with a local change in recombination that would alter *N*_e_ relative to other regions of the genome. Temporal variation in recombination landscapes, therefore, adds another layer of uncertainty for long-term *N*_e_ at a specific genomic location and can influence studies of selection that combine divergence (past) and diversity (present) data.

In general terms, frequent temporal changes in local recombination rates may generate evolutionary patterns that will closely follow the harmonic mean of *N*_e_ across generations and thus predict a tendency for past *N*_e_ and past *N*_e_ × *s* to be smaller than current *N*_e_ and *N*_e_ × *s*. As discussed in [79,116,119], a potential consequence of such temporal disparity in *N*_e_ × *s* is an excess of fixed weakly deleterious mutations relative to levels of diversity, a pattern that could be also interpreted as evidence for adaptive mutations under models that assume constant population size. Equivalently, if recombination rates change frequently, studies that use divergence data to estimate the distribution of selection coefficients on deleterious mutations will estimate past *N*_e_ × *s* and may underestimate recent selection and the consequences of BGS on diversity. Moving forward, analyses of selection may benefit from including the effects of demography [97,116] *and* from allowing for ‘demographic’ parameters to vary across genomes in order to capture the consequences of changes in recombination landscapes (see above). In addition, Smukowski Heil *et al*. [59] have proposed restricting analyses to regions that exhibit conserved recombination rates between species. To this end, further efforts should be invested in generating recombination data not only for the populations under study but also for outgroup populations and species, as is customary for evolutionary analyses using sequence data.

### (c) Influence of incomplete genome annotations

High-resolution predictions of BGS are as good as the genomic annotation used to assign the distribution of sites potentially targeted by deleterious mutations. Genomic annotations are, however, a work in progress and depend on both the methodology used to obtain data (e.g. transcriptomes) and the variety of biotic (e.g. cell types, age and sex) and abiotic (e.g. temperature and food) conditions investigated. The *D. melanogaster* genome annotation is a good case in point. Only 2 years after the initial genome reference was released (Release 1 [131]), the *D. melanogaster* Release 3 [132] altered the majority (85%) of gene models. Even more significantly, Release 5 (2006) of the genome annotation described almost 7000 new alternative splicing forms, 1200 novel ncRNAs and more than 1000 new genes when compared with Release 4 (2004) [133]. Predictably, genome annotations vary almost always in the direction of describing novel functional regions and reveal that a fraction of previously assumed neutral sites is, in fact, sporadically or constitutively under selection. To exemplify how this point can influence population genomic studies, I used *D. melanogaster* genome annotations from Release 4 (2004) and the more recent R6 (2016) [133]. The fraction of genomic sequence solely annotated as intergenic (not counting ‘N's) decreases from 0.40 to 0.27 for R4 and R6, respectively. The study of nucleotide diversity from the African Rwanda (RG) population of *D. melanogaster* [134,135] also reveals that the use of incomplete annotations can underestimate levels of diversity and generate a more negative Tajima's *D* at putatively neutral sites. Genome-wide levels of neutral diversity (fourfold synonymous sites; *π*_4f_) are substantially lower when using the R4 relative to when using the R6 annotation (median *π*_4f_ of 0.009 and 0.014, respectively). Similarly, the relative Tajima's *D* at fourfold synonymous sites is more negative when using R4 than when using the R6 annotation (median *D*/*D*_min_ of −0.101 and −0.011, respectively).

It follows that studies using an incomplete annotation may also underestimate the influence of BGS across genomes. To quantify this potential effect, I used the complete R4 and R6 genome annotations to generate two different, annotation-specific, high-resolution *B* landscapes under a BGS model [79]. At 100 kb scale, BGS generates *B* landscapes that fit substantially better the observed variation in *π*_4f_ across autosomes when using data from R6 (*R*^2^ = 0.484 and *ρ*^2^ = 0.557 for genomic regions with recombination rates greater than 0.1 cM per Mb) than when using the more incomplete annotation R4 (*R*^2^ = 0.226 and *ρ*^2^ = 0.358 for genomic regions with recombination rates greater than 0.1 cM per Mb). Equivalent conclusions are drawn for analyses at 1 and 10 kb scales (data not shown).

The reduction in predictive power of BGS to explain neutral diversity across the genome together with inaccurate estimates of neutral diversity when using an incomplete annotation uncovers limitations in studies of selection and linkage, particularly for non-model systems. Equivalent caveats may emerge from using partial information such as when considering only protein-coding sequences or when using a single transcript for genes with multiple transcripts. In all these cases, the influence of BGS could be underestimated and may therefore generate a tendency towards overestimating the need to include adaptive events. For model organisms such *D. melanogaster*, with a comprehensive annotation, a better alternative may be to follow a ‘shadowing’ approach where all annotations (coding and non-coding genes, all alternative splicing variants, TEs and repetitive sequences) are mapped onto a reference sequence, and only sites that are never annotated as being part of a functional region should be considered as potentially neutral [79].

### (d) Consequences of ignoring non-crossover (gene conversion) recombination events

Recombination results from the repair of DNA double-strand breaks through either CO events which shuffle large genomic regions between homologous chromosomes or NCO events which only involve the transfer of short genomic segments called gene conversion tracts. Gene conversion tracts are often only a few hundred nucleotides long or even shorter [40,136–139]; therefore, the number and genomic location of potential new allelic combinations caused by NCOs is highly limited relative to the overall effects associated with COs. As a consequence, NCO recombination has been often assumed to play a minor role in reducing hitchhiking effects in natural populations. In fact, most studies of selection and linkage directly omit NCOs and use COs as the only source of recombination when predicting patterns of diversity and effectiveness of selection across genomes.

The comparison of *B* landscapes based on BGS models that consider only COs (*B*_CO_) and those that include both CO and NCO events (*B*_CO+NCO_), however, reveals that *B*_CO+NCO_ landscapes perform consistently better than *B*_CO_ landscapes when describing variation in nucleotide diversity across the *D. melanogaster* genome [79]. This result is also in agreement with previous studies by Loewe & Charlesworth [50], and the more recent study by Campos *et al*. [140] showing that models of BGS that consider CO and NCO recombination can better explain evolutionary patterns across and among genes than models that consider only CO [49,50,141,142]. Combined, these results reveal a non-trivial influence of NCOs and the importance of using both COs and NCOs in studies of genetic hitchhiking and BGS specifically, at least for *Drosophila*. High-resolution maps of NCO rates are, however, still difficult to obtain. At a practical level, and because NCO rates show a more limited range of variation across genomes than CO rates [40,137,138], it may be sensible for future studies of selection and linkage to use variable CO rates together with a genome-wide average rate for NCOs when generating fine-scale landscapes under BGS or BGS + CS models.

### (e) Consequences of not considering balancing selection

An advantage to using a BGS prediction as baseline for levels of diversity across genomes is that it allows the detection of regions under modes of selection other than CS, such as balancing selection [79]. In *D. melanogaster*, a number of studies have revealed signatures of balancing selection associated with immunity genes and fitness-related temporal and spatial variation [19,79,86,143–145]. That is, our current understanding of selective forces acting in natural populations of this species suggests that the number of genes potentially associated with balancing selection may not be much smaller than that of genes showing clear signals of recent selective sweeps (or at least not smaller by orders of magnitude).

When balancing selection is not included in methods where diversity data are fitted to selection models, regions experiencing balancing selection (with an excess of diversity) are likely to be taken as regions with the weakest degree of genetic hitchhiking. Such a case would move upward genome-wide estimates of *B* (underestimate BGS), thus possibly leaving unexplained the regions with the lowest levels of diversity. This scenario may have little influence on genome-wide patterns of selection, but I propose that future studies designed to identify CS within a general BGS framework may benefit from first identifying and excluding from the analysis genomic regions with diversity-based signatures of balancing selection.

### (f) Influence of different methods for creating recombination maps

The direct analysis of co-inheritance of markers in meiotic products is the classic experimental approach for detecting recombination events. Advances in high-throughput sequencing and genotyping methods now allow the study of thousands of genetic markers (single nucleotide polymorphisms (SNPs) or small indels) and the generation of fine-scale maps of recombination events across genomes. Although this direct approach is still time-consuming and relatively expensive, whole-genome high-resolution recombination maps are available for a number of taxa, including yeast [137,146], humans [147–149], mice [150], dogs [151], *Drosophila* [40,41], *Caenorhabditis elegans* [152,153] and birds [154]. A complementary approach was proposed by Singh *et al*. [155], with a clever experimental and sequencing design that can generate recombination maps between two visual markers with unparalleled ultra-high resolution, thus adequate for gene-level studies across a specific genomic region. Moreover, when marker density is high enough, direct approaches can identify NCO and CO events [40,137,138]. In all, high-resolution experimental maps of recombination rates can be used to parametrize models of selection and linkage and are required to study the molecular basis of recombination plasticity (e.g. [156]).

On the potentially negative side, the genotyping strategies used in these experimental maps are almost certainly limited to a small number of genotypes and biotic/abiotic conditions; therefore, these recombination landscapes might differ, to an unknown degree, from those in natural populations. Moreover, the presence of polymorphic chromosomal inversions in the individuals used to create genetic maps could not only reduce CO rates within the inverted region, but also increase rates elsewhere in the genome (the interchromosomal effect [157,158]). Additionally, even with the new methodologies, it is unrealistic for most species to generate genome-wide recombination maps that provide reliable recombination rates at sub-gene resolution (≤10 kb), particularly when it is applied to multiple crosses and conditions. Furthermore, the degree of conservation of recombination rate within and between species decreases fast at finer scales (see [59] and references therein). All these factors are probably responsible—at least in part—for the strong dependence on the genomic window size of the goodness of fit between predictions of models of selection and observed diversity in all species analysed.

An alternative approach to obtaining recombination maps is to take advantage of whole-genome sequence data from multiple individuals and use linkage disequilibrium (LD) between polymorphisms to estimate recombination rate [128,159–165]. Based on population genetic theory, estimates of LD can be transformed into a population-scaled estimate of recombination (LD*ρ*; LD*ρ* ∼ *N*_e_ × *r*), where *r* is the recombination rate/bp/generation). This approach can be easily applied genome-wide and provides an estimate of historical recombination rates that are often at a much higher resolution than traditional cross- or pedigree-based genetic maps (see [166] for a review contrasting LD- and pedigree-based approaches to estimating recombination rates). A downside of the LD-based methods is that they require the use of an estimate of *N*_e_ to obtain the more relevant recombination rate *r*. This challenge may be significant because it may not always be direct to gauge the adequate *N*_e_ for a given genomic region (see above). Moreover, estimates of LD*ρ* generate a sex-average compound estimate of historical recombination that may not be adequate to study recent patterns of diversity owing to the potential change in recombination rates across genomes with time, including changes in the frequency of polymorphic chromosomal inversions (see above).

Below, however, I briefly discuss another potential challenge (to be described in more detail elsewhere). A number of the proposed methods for estimating LD*ρ* at genome-wide scale generate a compound estimate of CO plus NCO rates. This is relevant for at least two reasons. First, COs and NCOs play different roles in predicting the effects of selection at nearby sites and should be used separately in models of selection and linkage. Second, for population genomic analyses using high-density SNP data (as opposed to markers separated by hundreds of kilobases), the distance between SNPs can influence how much of the total LD*ρ* is due to NCOs. This is because most, if not all, COs will be detected regardless of the distance between the SNPs flanking the location of a CO, whereas the probability of detecting a gene conversion tract decreases with increasing distance between SNPs. Therefore, regions with high levels of nucleotide diversity (or marker density) will include more NCOs in estimates of LD*ρ* than those with lower levels of diversity. If this rationale is correct, reducing SNP density (‘thinning’ SNP data) should be accompanied by a reduction in estimates of LD*ρ* in species with high levels of nucleotide diversity. In agreement, Chan *et al*. [128] reported a reduction in estimates of LD*ρ* when they reduced SNP density: eliminating half of the SNPs along the X chromosome of the *D. melanogaster* RG population caused a moderate (13%) reduction in LD*ρ*. To further investigate this trend, I applied a more extreme thinning strategy to the same RG population by using 1 of every 10 informative SNPs, increasing the average distance between SNPs to approximately 400 bp. LD*ρ* estimates using low SNP density (LD*ρ*_SNP1/10_) are severely reduced relative to those estimated when using the complete SNP dataset (median LD*ρ* of 0.011 and 0.021, respectively; Wilcoxon matched pairs test *Z* = 20.7, *p* < 1 × 10^−100^ based on 100 kb non-overlapping regions).

Thus, estimates of LD*ρ* may vary across genomes not only due to differences in CO and NCO rates, but also as a result of a higher fraction of NCOs that will be detected when SNP density increases. The potential upward bias in LD*ρ* in regions with high nucleotide diversity is particularly relevant in studies of selection and linkage, because a positive relationship between recombination rates and levels of diversity is accepted as evidence of pervasive selection within BGS and CS/Draft models. In short, in species with high levels of diversity or with variable mutation rates across genomes, the use of recombination rates based on LD*ρ* could overestimate the impact of selection on nearby diversity.

Computational demands are still limiting genome-wide applications of full-likelihood methods for jointly estimating CO and NCO rates from population genomic data [167]. Meanwhile, I propose considering the influence of variable SNP density in LD-based estimates of recombination that do not differentiate between COs and NCOs. Controlled thinning strategies may help to identify and lessen potential biases in species, or genomic regions, with high levels of nucleotide diversity.

## 4. Conclusion

Here, I reviewed the main models of selection and linkage applicable to recombining genomes and two studies supporting the concept that BGS explains a very large fraction of the variation in diversity across the whole genome in *D. melanogaster*. As such, the BGS framework should be accepted as a sensible null model to study other forms of natural selection. I also identified and discussed demographic, analytical and methodological challenges in studies of selection at linked sites that have been often overlooked. Some of the challenges are easily addressable and I put forward that all should be considered when designing future studies of selection. Notably, several of these challenges and limitations share a potential bias towards overestimating the evidence supporting recent selective sweeps to the detriment of a BGS explanation. In part, some of the challenges stem from the units of reliable data. Most, if not all, selective sweeps initially identified in *D. melanogaster* covered large genomic regions of tens or hundreds of kilobases and continue to be fine examples of recent adaptive events. Inaccurate recombination rates at the scale of single genes or incomplete genome annotations have evolutionary consequences at finer scales, and caution should be applied when inferring selective signals at this resolution.

From several perspectives, the effects of selection on linked sites could be regarded by population geneticists as equivalent to a physicist's view of gravitational waves (though—understandably—with much less fanfare on the news and popular culture). The analogy of selection disrupting the dynamics of drift-related parameters around selected sites, altering (curving) *N*_e_ along chromosomes, may be—up to a point—not merely a graphical one but also one that exemplifies how insightful theoretical works move research forward. As discussed, current approaches to identify the signatures of selection using diversity data across genomes do not fully consider the joint effects of demography, genetic linkage, rapid temporal changes in recombination landscapes and different forms of selection. The next steps towards a better understanding of how all these factors influence different genomic regions may require combining traditional population genetics, forward simulations and machine learning methods.

## Supplementary Material

Population dynamics under varying intensities of BGS.

## Data accessibility

This article has no additional data.

## References

[1] Charlesworth B, Morgan MT, Charlesworth D 1993 The effect of deleterious mutations on neutral molecular variation. Genetics 134, 1289–1303. 8375663 10.1093/genetics/134.4.1289 PMC1205596

[2] Hudson RR, Kaplan NL 1995 Deleterious background selection with recombination. Genetics 141, 1605–1617. 8601498 10.1093/genetics/141.4.1605 PMC1206891

[3] Charlesworth B 1996 Background selection and patterns of genetic diversity in *Drosophila melanogaster*. Genet. Res. 68, 131–149. (10.1017/S0016672300034029) 8940902

[4] Nordborg M, Charlesworth B, Charlesworth D 1996 The effect of recombination on background selection. Genet. Res. 67, 159–174. (10.1017/S0016672300033619) 8801188

[5] Fisher RA 1930 The genetical theory of natural selection. New York, NY: Oxford University Press.

[6] Muller HJ 1932 Some genetic aspects of sex. Am. Nat. 66, 118–138. (10.1086/280418)

[7] Hill WG, Robertson A 1966 The effect of linkage on limits to artificial selection. Genet. Res. 8, 269–294. (10.1017/S0016672300010156) 5980116

[8] Felsenstein J 1974 The evolutionary advantage of recombination. Genetics 78, 737–756. 4448362 10.1093/genetics/78.2.737 PMC1213231

[9] Birky CW Jr, Walsh JB 1988 Effects of linkage on rates of molecular evolution. Proc. Natl Acad. Sci. USA 85, 6414–6418. (10.1073/pnas.85.17.6414) 3413105 PMC281982

[10] Kimura M 1962 On the probability of fixation of mutant genes in a population. Genetics 47, 713. 14456043 10.1093/genetics/47.6.713 PMC1210364

[11] Fisher RA 1930 The distribution of gene ratios for rare mutations. Proc. R. Soc. Edinb. 50, 205–220.

[12] Wright S 1931 Evolution in Mendelian populations. Genetics 16, 97–159. 17246615 10.1093/genetics/16.2.97 PMC1201091

[13] Maynard Smith J, Haigh J 1974 The hitch-hiking effect of a favorable gene. Genet. Res. 23, 23–35. (10.1017/S0016672300014634) 4407212

[14] Kim Y, Stephan W 2002 Detecting a local signature of genetic hitchhiking along a recombining chromosome. Genetics 160, 765–777. 11861577 10.1093/genetics/160.2.765 PMC1461968

[15] Wiehe TH, Stephan W 1993 Analysis of a genetic hitchhiking model, and its application to DNA polymorphism data from *Drosophila melanogaster*. Mol. Biol. Evol. 10, 842–854. 8355603 10.1093/oxfordjournals.molbev.a040046

[16] Gillespie JH 2000 Genetic drift in an infinite population. The pseudohitchhiking model. Genetics 155, 909–919. 10835409 10.1093/genetics/155.2.909 PMC1461093

[17] Hudson RR, Kaplan NL 1994 Gene trees with background selection. In Non-neutral evolution: theories and molecular data (ed. Brian G), pp. 140–153. New York, NY: Chapman & Hall.

[18] Strobeck C 1983 Expected linkage disequilibrium for a neutral locus linked to a chromosomal arrangement. Genetics 103, 545–555. 6404695 10.1093/genetics/103.3.545 PMC1202039

[19] Hudson RR, Kreitman M, Aguade M 1987 A test of neutral molecular evolution based on nucleotide data. Genetics 116, 153–159. 3110004 10.1093/genetics/116.1.153 PMC1203113

[20] Hudson RR, Kaplan NL 1988 The coalescent process in models with selection and recombination. Genetics 120, 831–840. 3147214 10.1093/genetics/120.3.831 PMC1203560

[21] Charlesworth B, Nordborg M, Charlesworth D 1997 The effects of local selection, balanced polymorphism and background selection on equilibrium patterns of genetic diversity in subdivided populations. Genet. Res. 70, 155–174. (10.1017/S0016672397002954) 9449192

[22] Takahata N, Satta Y 1998 Footprints of intragenic recombination at HLA loci. Immunogenetics 47, 430–441. (10.1007/s002510050380) 9553149

[23] Charlesworth D 2006 Balancing selection and its effects on sequences in nearby genome regions. PLoS Genet. 2, e64 (10.1371/journal.pgen.0020064) 16683038 PMC1449905

[24] Sved JA 1968 The stability of linked systems of loci with a small population size. Genetics 59, 543–563. 5708301 10.1093/genetics/59.4.543 PMC1212022

[25] Zhao L, Charlesworth B 2016 Resolving the conflict between associative overdominance and background selection. Genetics 203, 1315–1334. (10.1534/genetics.116.188912) 27182952 PMC4937488

[26] Ota T 1971 Associative overdominance caused by linked detrimental mutations. Genet. Res. 18, 277–286. (10.1017/S0016672300012684) 5158298

[27] Ohta T, Kimura M 1970 Development of associative overdominance through linkage disequilibrium in finite populations. Genet. Res. 16, 165–177. (10.1017/S0016672300002391) 5516427

[28] Aguade M, Miyashita N, Langley CH 1989 Restriction-map variation at the *zeste*-*tko* region in natural populations of *Drosophila melanogaster*. Mol. Biol. Evol. 6, 123–130. 2566105 10.1093/oxfordjournals.molbev.a040538

[29] Stephan W, Langley CH 1989 Molecular genetic variation in the centromeric region of the X chromosome in three *Drosophila ananassae* populations. I. Contrasts between the *vermilion* and *forked* loci. Genetics 121, 89–99. 2563714 10.1093/genetics/121.1.89 PMC1203608

[30] Begun DJ, Aquadro CF 1992 Levels of naturally occurring DNA polymorphism correlate with recombination rates in *D. melanogaster*. Nature 356, 519–520. (10.1038/356519a0) 1560824

[31] Aguadé M, Langley CH 1994 Polymorphism and divergence in regions of low recombination in *Drosophila*. In Non-neutral evolution: theories and molecular data (ed. Golding B), pp. 67–76. New York, NY: Chapman & Hall.

[32] Aquadro CF, Begun DJ, Kindahl EC 1994 Selection, recombination and DNA polymorphism in *Drosophila*. In Non-neutral evolution: theories and molecular data (ed. Golding B), pp. 46–56. New York, NY: Chapman & Hall.

[33] Charlesworth B, Campos JL 2014 The relations between recombination rate and patterns of molecular variation and evolution in *Drosophila*. Annu. Rev. Genet. 48, 383–403. (10.1146/annurev-genet-120213-092525) 25251853

[34] Nachman MW, Bauer VL, Crowell SL, Aquadro CF 1998 DNA variability and recombination rates at X-linked loci in humans. Genetics 150, 1133–1141. 9799265 10.1093/genetics/150.3.1133 PMC1460397

[35] Payseur BA, Nachman MW 2002 Gene density and human nucleotide polymorphism. Mol. Biol. Evol. 19, 336–340. (10.1093/oxfordjournals.molbev.a004086) 11861892

[36] McVicker G, Gordon D, Davis C, Green P 2009 Widespread genomic signatures of natural selection in hominid evolution. PLoS Genet. 5, e1000471 (10.1371/journal.pgen.1000471) 19424416 PMC2669884

[37] Cutter AD, Choi JY 2010 Natural selection shapes nucleotide polymorphism across the genome of the nematode *Caenorhabditis briggsae*. Genome Res. 20, 1103–1111. (10.1101/gr.104331.109) 20508143 PMC2909573

[38] Cutter AD, Moses AM 2011 Polymorphism, divergence, and the role of recombination in *Saccharomyces cerevisiae* genome evolution. Mol. Biol. Evol. 28, 1745–1754. (10.1093/molbev/msq356) 21199893

[39] Lohmueller KE et al. 2011 Natural selection affects multiple aspects of genetic variation at putatively neutral sites across the human genome. PLoS Genet. 7, e1002326 (10.1371/journal.pgen.1002326) 22022285 PMC3192825

[40] Comeron JM, Ratnappan R, Bailin S 2012 The many landscapes of recombination in *Drosophila melanogaster*. PLoS Genet. 8, e1002905 (10.1371/journal.pgen.1002905) 23071443 PMC3469467

[41] McGaugh SE, Heil CS, Manzano-Winkler B, Loewe L, Goldstein S, Himmel TL, Noor MA 2012 Recombination modulates how selection affects linked sites in *Drosophila*. PLoS Biol. 10, e1001422 (10.1371/journal.pbio.1001422) 23152720 PMC3496668

[42] Flowers JM, Molina J, Rubinstein S, Huang P, Schaal BA, Purugganan MD 2012 Natural selection in gene-dense regions shapes the genomic pattern of polymorphism in wild and domesticated rice. Mol. Biol. Evol. 29, 675–687. (10.1093/molbev/msr225) 21917724

[43] Nachman MW, Payseur BA 2012 Recombination rate variation and speciation: theoretical predictions and empirical results from rabbits and mice. Phil. Trans. R. Soc. B 367, 409–421. (10.1098/rstb.2011.0249) 22201170 PMC3233716

[44] Cutter AD, Payseur BA 2013 Genomic signatures of selection at linked sites: unifying the disparity among species. Nat. Rev. Genet. 14, 262–274. (10.1038/nrg3425) 23478346 PMC4066956

[45] Martin SH, Most M, Palmer WJ, Salazar C, McMillan WO, Jiggins FM, Jiggins CD 2016 Natural selection and genetic diversity in the butterfly *Heliconius melpomene*. Genetics 203, 525–541. (10.1534/genetics.115.183285) 27017626 PMC4858797

[46] Hey J, Kliman RM 2002 Interactions between natural selection, recombination and gene density in the genes of *Drosophila*. Genetics 160, 595. 11861564 10.1093/genetics/160.2.595 PMC1461979

[47] Comeron JM, Kreitman M, Aguade M 1999 Natural selection on synonymous sites is correlated with gene length and recombination in *Drosophila*. Genetics 151, 239–249. 9872963 10.1093/genetics/151.1.239 PMC1460462

[48] Comeron JM, Kreitman M 2000 The correlation between intron length and recombination in *Drosophila*. Dynamic equilibrium between mutational and selective forces. Genetics 156, 1175–1190. 11063693 10.1093/genetics/156.3.1175 PMC1461334

[49] Comeron JM, Kreitman M 2002 Population, evolutionary and genomic consequences of interference selection. Genetics 161, 389–410. 12019253 10.1093/genetics/161.1.389 PMC1462104

[50] Loewe L, Charlesworth B 2007 Background selection in single genes may explain patterns of codon bias. Genetics 175, 1381–1393. (10.1534/genetics.106.065557) 17194784 PMC1840058

[51] Messer PW, Petrov DA 2013 Population genomics of rapid adaptation by soft selective sweeps. Trends Ecol. Evol. 28, 659–669. (10.1016/j.tree.2013.08.003) 24075201 PMC3834262

[52] Santiago E, Caballero A 2016 Joint prediction of the effective population size and the rate of fixation of deleterious mutations. Genetics 204, 1267–1279. (10.1534/genetics.116.188250) 27672094 PMC5105856

[53] Masel J 2012 Rethinking Hardy–Weinberg and genetic drift in undergraduate biology. BioEssays 34, 701–710. (10.1002/bies.201100178) 22576789

[54] Charlesworth B 2009 Fundamental concepts in genetics: effective population size and patterns of molecular evolution and variation. Nat. Rev. Genet. 10, 195–205. (10.1038/nrg2526) 19204717

[55] Gillespie JH 2001 Is the population size of a species relevant to its evolution? Evolution 55, 2161–2169. (10.1111/j.0014-3820.2001.tb00732.x) 11794777

[56] Neher RA, Shraiman BI 2011 Genetic draft and quasi-neutrality in large facultatively sexual populations. Genetics 188, 975–996. (10.1534/genetics.111.128876) 21625002 PMC3176096

[57] Johnson T, Barton NH 2002 The effect of deleterious alleles on adaptation in asexual populations. Genetics 162, 395–411. 12242249 10.1093/genetics/162.1.395 PMC1462245

[58] Stevison LS et al. 2016 The time scale of recombination rate evolution in great apes. Mol. Biol. Evol. 33, 928–945. (10.1093/molbev/msv331) 26671457 PMC5870646

[59] Smukowski Heil CS, Ellison C, Dubin M, Noor MA 2015 Recombining without hotspots: a comprehensive evolutionary portrait of recombination in two closely related species of *Drosophila*. Genome Biol. Evol. 7, 2829–2842. (10.1093/gbe/evv182) 26430062 PMC4684701

[60] Jensen-Seaman MI, Furey TS, Payseur BA, Lu Y, Roskin KM, Chen CF, Thomas MA, Haussler D, Jacob HJ 2004 Comparative recombination rates in the rat, mouse, and human genomes. Genome Res. 14, 528–538. (10.1101/gr.1970304) 15059993 PMC383296

[61] Singhal S et al. 2015 Stable recombination hotspots in birds. Science 350, 928–932. (10.1126/science.aad0843) 26586757 PMC4864528

[62] Lam I, Keeney S 2015 Nonparadoxical evolutionary stability of the recombination initiation landscape in yeast. Science 350, 932–937. (10.1126/science.aad0814) 26586758 PMC4656144

[63] Kaiser VB, Charlesworth B 2009 The effects of deleterious mutations on evolution in non-recombining genomes. Trends Genet. 25, 9–12. (10.1016/j.tig.2008.10.009) 19027982

[64] Good BH, Walczak AM, Neher RA, Desai MM 2014 Genetic diversity in the interference selection limit. PLoS Genet. 10, e1004222 (10.1371/journal.pgen.1004222) 24675740 PMC3967937

[65] Good BH, Rouzine IM, Balick DJ, Hallatschek O, Desai MM 2012 Distribution of fixed beneficial mutations and the rate of adaptation in asexual populations. Proc. Natl Acad. Sci. USA 109, 4950–4955. (10.1073/pnas.1119910109) 22371564 PMC3323973

[66] Weissman DB, Barton NH 2012 Limits to the rate of adaptive substitution in sexual populations. PLoS Genet. 8, e1002740 (10.1371/journal.pgen.1002740) 22685419 PMC3369949

[67] Neher RA 2013 Genetic draft, selective interference, and population genetics of rapid adaptation. Annu. Rev. Ecol. Evol. Syst. 44, 195–215. (10.1146/annurev-ecolsys-110512-135920)

[68] Kaiser VB, Charlesworth B 2010 Muller's ratchet and the degeneration of the *Drosophila miranda* neo-Y chromosome. Genetics 185, 339–348. (10.1534/genetics.109.112789) 20215466 PMC2870968

[69] Muller HJ 1964 The relation of recombination to mutational advance. Mutat. Res. 1, 2–9. (10.1016/0027-5107(64)90047-8) 14195748

[70] Gordo I, Navarro A, Charlesworth B 2002 Muller's ratchet and the pattern of variation at a neutral locus. Genetics 161, 835–848. 12072478 10.1093/genetics/161.2.835 PMC1462134

[71] Loewe L 2006 Quantifying the genomic decay paradox due to Muller's ratchet in human mitochondrial DNA. Genet. Res. 87, 133–159. (10.1017/S0016672306008123) 16709275

[72] Rouzine IM, Brunet E, Wilke CO 2008 The traveling-wave approach to asexual evolution: Muller's ratchet and speed of adaptation. Theor. Popul. Biol. 73, 24–46. (10.1016/j.tpb.2007.10.004) 18023832 PMC2246079

[73] Loewe L, Hill WG 2010 The population genetics of mutations: good, bad and indifferent. Phil. Trans. R. Soc. B 365, 1153–1167. (10.1098/rstb.2009.0317) 20308090 PMC2871823

[74] Neher RA, Shraiman BI 2012 Fluctuations of fitness distributions and the rate of Muller's ratchet. Genetics 191, 1283–1293. (10.1534/genetics.112.141325) 22649084 PMC3416007

[75] Muller HJ 1950 Our load of mutations. Am. J. Hum. Genet. 2, 111–176. 14771033 PMC1716299

[76] Kimura M 1983 The neutral theory of molecular evolution. Cambridge, UK: Cambridge University Press.

[77] Ohta T 1992 The nearly neutral theory of molecular evolution. Annu. Rev. Ecol. Syst. 23, 263–286. (10.1146/annurev.es.23.110192.001403)

[78] Ohta T 1976 Role of very slightly deleterious mutations in molecular evolution and polymorphism. Theor. Popul. Biol. 10, 254–275. (10.1016/0040-5809(76)90019-8) 1013905

[79] Comeron JM 2014 Background selection as baseline for nucleotide variation across the *Drosophila* genome. PLoS Genet. 10, e1004434 (10.1371/journal.pgen.1004434) 24968283 PMC4072542

[80] Phung TN, Huber CD, Lohmueller KE 2016 Determining the effect of natural selection on linked neutral divergence across species. PLoS Genet. 12, e1006199 (10.1371/journal.pgen.1006199) 27508305 PMC4980041

[81] Pfeifer SP, Jensen JD 2016 The impact of linked selection in chimpanzees: a comparative study. Genome Biol. Evol. 8, 3202–3208. (10.1093/gbe/evw240) 27678122 PMC5174744

[82] Elyashiv E, Sattath S, Hu TT, Strutsovsky A, McVicker G, Andolfatto P, Coop G, Sella G 2016 A genomic map of the effects of linked selection in *Drosophila*. PLoS Genet. 12, e1006130 (10.1371/journal.pgen.1006130) 27536991 PMC4990265

[83] Charlesworth B 2012 The role of background selection in shaping patterns of molecular evolution and variation: evidence from variability on the *Drosophila* X chromosome. Genetics 191, 233–246. (10.1534/genetics.111.138073) 22377629 PMC3338263

[84] Reed FA, Akey JM, Aquadro CF 2005 Fitting background-selection predictions to levels of nucleotide variation and divergence along the human autosomes. Genome Res. 15, 1211–1221. (10.1101/gr.3413205) 16140989 PMC1199535

[85] Sella G, Petrov DA, Przeworski M, Andolfatto P 2009 Pervasive natural selection in the *Drosophila* genome? PLoS Genet. 5, e1000495 (10.1371/journal.pgen.1000495) 19503600 PMC2684638

[86] Langley CH et al. 2012 Genomic variation in natural populations of *Drosophila melanogaster*. Genetics 192, 533–598. (10.1534/genetics.112.142018) 22673804 PMC3454882

[87] Jensen JD, Thornton KR, Andolfatto P 2008 An approximate Bayesian estimator suggests strong, recurrent selective sweeps in *Drosophila*. PLoS Genet. 4, e1000198 (10.1371/journal.pgen.1000198) 18802463 PMC2529407

[88] Jensen JD, Bauer DuMont VL, Ashmore AB, Gutierrez A, Aquadro CF 2007 Patterns of sequence variability and divergence at the diminutive gene region of *Drosophila melanogaster*: complex patterns suggest an ancestral selective sweep. Genetics 177, 1071–1085. (10.1534/genetics.106.069468) 17720938 PMC2034614

[89] DuMont VB, Aquadro CF 2005 Multiple signatures of positive selection downstream of *Notch* on the X chromosome in *Drosophila melanogaster*. Genetics 171, 639–653. (10.1534/genetics.104.038851) 16020794 PMC1456778

[90] Svetec N, Pavlidis P, Stephan W 2009 Recent strong positive selection on *Drosophila melanogaster* HDAC6, a gene encoding a stress surveillance factor, as revealed by population genomic analysis. Mol. Biol. Evol. 26, 1549–1556. (10.1093/molbev/msp065) 19349642

[91] Beisswanger S, Stephan W, De Lorenzo D 2006 Evidence for a selective sweep in the *wapl* region of *Drosophila melanogaster*. Genetics 172, 265–274. (10.1534/genetics.105.049346) 16204208 PMC1456153

[92] Fu YX 1997 Statistical tests of neutrality of mutations against population growth, hitchhiking and background selection. Genetics 147, 915–925. 9335623 10.1093/genetics/147.2.915 PMC1208208

[93] Zeng K, Fu YX, Shi S, Wu CI 2006 Statistical tests for detecting positive selection by utilizing high-frequency variants. Genetics 174, 1431–1439. (10.1534/genetics.106.061432) 16951063 PMC1667063

[94] Zeng K, Mano S, Shi S, Wu CI 2007 Comparisons of site- and haplotype-frequency methods for detecting positive selection. Mol. Biol. Evol. 24, 1562–1574. (10.1093/molbev/msm078) 17449894

[95] Charlesworth D, Charlesworth B, Morgan MT 1995 The pattern of neutral molecular variation under the background selection model. Genetics 141, 1619–1632. 8601499 10.1093/genetics/141.4.1619 PMC1206892

[96] Tajima F 1989 Statistical method for testing the neutral mutation hypothesis by DNA polymorphism. Genetics 123, 585–595. 2513255 10.1093/genetics/123.3.585 PMC1203831

[97] Zeng K, Charlesworth B 2011 The joint effects of background selection and genetic recombination on local gene genealogies. Genetics 189, 251–266. (10.1534/genetics.111.130575) 21705759 PMC3176134

[98] Nicolaisen LE, Desai MM 2013 Distortions in genealogies due to purifying selection and recombination. Genetics 195, 221–230. (10.1534/genetics.113.152983) 23821597 PMC3761303

[99] Walczak AM, Nicolaisen LE, Plotkin JB, Desai MM 2012 The structure of genealogies in the presence of purifying selection: a fitness-class coalescent. Genetics 190, 753–779. (10.1534/genetics.111.134544) 22135349 PMC3276618

[100] Ewing GB, Jensen JD 2016 The consequences of not accounting for background selection in demographic inference. Mol. Ecol. 25, 135–141. (10.1111/mec.13390) 26394805

[101] Huber CD, DeGiorgio M, Hellmann I, Nielsen R 2016 Detecting recent selective sweeps while controlling for mutation rate and background selection. Mol. Ecol. 25, 142–156. (10.1111/mec.13351) 26290347 PMC5082542

[102] Zeng K, Corcoran P 2015 The effects of background and interference selection on patterns of genetic variation in subdivided populations. Genetics 201, 1539–1554. (10.1534/genetics.115.178558) 26434720 PMC4676517

[103] Elyashiv E, Bullaughey K, Sattath S, Rinott Y, Przeworski M, Sella G 2010 Shifts in the intensity of purifying selection: an analysis of genome-wide polymorphism data from two closely related yeast species. Genome Res. 20, 1558–1573. (10.1101/gr.108993.110) 20817943 PMC2963819

[104] Beissinger TM, Wang L, Crosby K, Durvasula A, Hufford MB, Ross-Ibarra J 2016 Recent demography drives changes in linked selection across the maize genome. Nat. Plants 2, 16084 (10.1038/nplants.2016.84) 27294617

[105] Zeng K, Charlesworth B 2010 The effects of demography and linkage on the estimation of selection and mutation parameters. Genetics 186, 1411–1424. (10.1534/genetics.110.122150) 20923980 PMC2998320

[106] Zeng K 2013 A coalescent model of background selection with recombination, demography and variation in selection coefficients. Heredity 110, 363–371. (10.1038/hdy.2012.102) 23188176 PMC3607694

[107] Wall JD, Andolfatto P, Przeworski M 2002 Testing models of selection and demography in *Drosophila simulans*. Genetics 162, 203–216. 12242234 10.1093/genetics/162.1.203 PMC1462246

[108] Pool JE, Nielsen R 2007 Population size changes reshape genomic patterns of diversity. Evolution 61, 3001–3006. (10.1111/j.1558-5646.2007.00238.x) 17971168 PMC3443680

[109] Keinan A, Mullikin JC, Patterson N, Reich D 2009 Accelerated genetic drift on chromosome X during the human dispersal out of Africa. Nat. Genet. 41, 66–70. (10.1038/ng.303) 19098910 PMC2612098

[110] Campos JL, Halligan DL, Haddrill PR, Charlesworth B 2014 The relation between recombination rate and patterns of molecular evolution and variation in *Drosophila melanogaster*. Mol. Biol. Evol. 31, 1010–1028. (10.1093/molbev/msu056) 24489114 PMC3969569

[111] Fay JC, Wu CI 1999 A human population bottleneck can account for the discordance between patterns of mitochondrial versus nuclear DNA variation. Mol. Biol. Evol. 16, 1003–1005. (10.1093/oxfordjournals.molbev.a026175) 10406117

[112] Hey J, Harris E 1999 Population bottlenecks and patterns of human polymorphism. Mol. Biol. Evol. 16, 1423–1426. (10.1093/oxfordjournals.molbev.a026054) 10563023

[113] Schaeffer SW 2002 Molecular population genetics of sequence length diversity in the *Adh* region of *Drosophila pseudoobscura*. Genet. Res. 80, 163–175. (10.1017/S0016672302005955) 12688655

[114] Fay JC, Wyckoff GJ, Wu CI 2001 Positive and negative selection on the human genome. Genetics 158, 1227–1234. 11454770 10.1093/genetics/158.3.1227 PMC1461725

[115] Smith NG, Eyre-Walker A 2002 Adaptive protein evolution in *Drosophila*. Nature 415, 1022–1024. (10.1038/4151022a) 11875568

[116] Eyre-Walker A, Keightley PD 2009 Estimating the rate of adaptive molecular evolution in the presence of slightly deleterious mutations and population size change. Mol. Biol. Evol. 26, 2097–2108. (10.1093/molbev/msp119) 19535738

[117] Keightley PD, Eyre-Walker A 2007 Joint inference of the distribution of fitness effects of deleterious mutations and population demography based on nucleotide polymorphism frequencies. Genetics 177, 2251–2261. (10.1534/genetics.107.080663) 18073430 PMC2219502

[118] Messer PW 2013 SLiM: simulating evolution with selection and linkage. Genetics 194, 1037–1039. (10.1534/genetics.113.152181) 23709637 PMC3730910

[119] Eyre-Walker A 2002 Changing effective population size and the McDonald–Kreitman test. Genetics 162, 2017–2024. 12524367 10.1093/genetics/162.4.2017 PMC1462352

[120] Haller BC, Messer PW 2017 SLiM 2: flexible, interactive forward genetic simulations. Mol. Biol. Evol. 34, 230–240. (10.1093/molbev/msw211) 27702775

[121] Messer PW, Petrov DA 2013 Frequent adaptation and the McDonald–Kreitman test. Proc. Natl Acad. Sci. USA 110, 8615–8620. (10.1073/pnas.1220835110) 23650353 PMC3666677

[122] Hernandez RD 2008 A flexible forward simulator for populations subject to selection and demography. Bioinformatics 24, 2786–2787. (10.1093/bioinformatics/btn522) 18842601 PMC2639268

[123] Hernandez RD, Uricchio LH 2015 SFS_CODE: more efficient and flexible forward simulations. bioRχiv (10.1101/025064)

[124] Schrider DR, Kern AD 2016 S/HIC: robust identification of soft and hard sweeps using machine learning. PLoS Genet. 12, e1005928 (10.1371/journal.pgen.1005928) 26977894 PMC4792382

[125] Schrider DR, Shanku AG, Kern AD 2016 Effects of linked selective sweeps on demographic inference and model selection. Genetics 204, 1207–1223. (10.1534/genetics.116.190223) 27605051 PMC5105852

[126] Sheehan S, Song YS 2016 Deep learning for population genetic inference. PLoS Comput. Biol. 12, e1004845 (10.1371/journal.pcbi.1004845) 27018908 PMC4809617

[127] Smukowski CS, Noor MA 2011 Recombination rate variation in closely related species. Heredity 107, 496–508. (10.1038/hdy.2011.44) 21673743 PMC3242630

[128] Chan AH, Jenkins PA, Song YS 2012 Genome-wide fine-scale recombination rate variation in *Drosophila melanogaster*. PLoS Genet. 8, e1003090 (10.1371/journal.pgen.1003090) 23284288 PMC3527307

[129] Dumont BL, White MA, Steffy B, Wiltshire T, Payseur BA 2011 Extensive recombination rate variation in the house mouse species complex inferred from genetic linkage maps. Genome Res. 21, 114–125. (10.1101/gr.111252.110) 20978138 PMC3012918

[130] Ptak SE, Hinds DA, Koehler K, Nickel B, Patil N, Ballinger DG, Przeworski M, Frazer KA, Paabo S 2005 Fine-scale recombination patterns differ between chimpanzees and humans. Nat. Genet. 37, 429–434. (10.1038/ng1529) 15723063

[131] Adams M et al. 2000 The genome sequence of *Drosophila melanogaster*. Science 287, 2185–2195. (10.1126/science.287.5461.2185) 10731132

[132] Misra S et al. 2002 Annotation of the *Drosophila melanogaster* euchromatic genome: a systematic review. Genome Biol. 3, RESEARCH0083 (10.1186/gb-2002-3-12-research0083) 12537572 PMC151185

[133] Hoskins RA et al. 2015 The release 6 reference sequence of the *Drosophila melanogaster* genome. Genome Res. 25, 445–458. (10.1101/gr.185579.114) 25589440 PMC4352887

[134] Lack JB, Cardeno CM, Crepeau MW, Taylor W, Corbett-Detig RB, Stevens KA, Langley CH, Pool JE 2015 The *Drosophila* genome nexus: a population genomic resource of 623 *Drosophila melanogaster* genomes, including 197 from a single ancestral range population. Genetics 199, 1229–1241. (10.1534/genetics.115.174664) 25631317 PMC4391556

[135] Pool JE et al. 2012 Population genomics of sub-Saharan *Drosophila melanogaster*: African diversity and non-African admixture. PLoS Genet. 8, e1003080 (10.1371/journal.pgen.1003080) 23284287 PMC3527209

[136] Hilliker AJ, Harauz G, Reaume AG, Gray M, Clark SH, Chovnick A 1994 Meiotic gene conversion tract length distribution within the *rosy* locus of *Drosophila melanogaster*. Genetics 137, 1019–1026. 7982556 10.1093/genetics/137.4.1019 PMC1206049

[137] Mancera E, Bourgon R, Brozzi A, Huber W, Steinmetz LM 2008 High-resolution mapping of meiotic crossovers and non-crossovers in yeast. Nature 454, 479–485. (10.1038/nature07135) 18615017 PMC2780006

[138] Miller DE, Smith CB, Kazemi NY, Cockrell AJ, Arvanitakas AV, Blumenstiel JP, Jaspersen SL, Hawley RS 2016 Whole-genome analysis of individual meiotic events in *Drosophila melanogaster* reveals that noncrossover gene conversions are insensitive to interference and the centromere effect. Genetics 203, 159–171. (10.1534/genetics.115.186486) 26944917 PMC4858771

[139] Jeffreys AJ, May CA 2004 Intense and highly localized gene conversion activity in human meiotic crossover hot spots. Nat. Genet. 36, 151–156. (10.1038/ng1287) 14704667

[140] Campos JL, Zhao L, Charlesworth B 2017 Estimating the parameters of background selection and selective sweeps in *Drosophila* in the presence of gene conversion. Proc. Natl Acad. Sci. USA 114, E4762–E4771. (10.1073/pnas.1619434114) 28559322 PMC5474792

[141] Comeron JM, Guthrie TB 2005 Intragenic Hill–Robertson interference influences selection intensity on synonymous mutations in *Drosophila*. Mol. Biol. Evol. 22, 2519–2530. (10.1093/molbev/msi246) 16120803

[142] Qin H, Wu WB, Comeron JM, Kreitman M, Li WH 2004 Intragenic spatial patterns of codon usage bias in prokaryotic and eukaryotic genomes. Genetics 168, 2245–2260. (10.1534/genetics.104.030866) 15611189 PMC1448744

[143] Croze M, Wollstein A, Bozicevic V, Zivkovic D, Stephan W, Hutter S 2017 A genome-wide scan for genes under balancing selection in *Drosophila melanogaster*. BMC Evol. Biol. 17, 15 (10.1186/s12862-016-0857-z) 28086750 PMC5237213

[144] Unckless RL, Lazzaro BP 2016 The potential for adaptive maintenance of diversity in insect antimicrobial peptides. Phil. Trans. R. Soc. B 371, 20150291 (10.1098/rstb.2015.0291) 27160594 PMC4874389

[145] Bergland AO, Behrman EL, O'Brien KR, Schmidt PS, Petrov DA 2014 Genomic evidence of rapid and stable adaptive oscillations over seasonal time scales in *Drosophila*. PLoS Genet. 10, e1004775 (10.1371/journal.pgen.1004775) 25375361 PMC4222749

[146] Pan J et al. 2011 A hierarchical combination of factors shapes the genome-wide topography of yeast meiotic recombination initiation. Cell 144, 719–731. (10.1016/j.cell.2011.02.009) 21376234 PMC3063416

[147] Myers S, Bottolo L, Freeman C, McVean G, Donnelly P 2005 A fine-scale map of recombination rates and hotspots across the human genome. Science 310, 321–324. (10.1126/science.1117196) 16224025

[148] Hinch AG et al. 2011 The landscape of recombination in African Americans. Nature 476, 170–175. (10.1038/nature10336) 21775986 PMC3154982

[149] Kong A et al. 2010 Fine-scale recombination rate differences between sexes, populations and individuals. Nature 467, 1099–1103. (10.1038/nature09525) 20981099

[150] Smagulova F, Gregoretti IV, Brick K, Khil P, Camerini-Otero RD, Petukhova GV 2011 Genome-wide analysis reveals novel molecular features of mouse recombination hotspots. Nature 472, 375–378. (10.1038/nature09869) 21460839 PMC3117304

[151] Campbell CL, Bherer C, Morrow BE, Boyko AR, Auton A 2016 A pedigree-based map of recombination in the domestic dog genome. G3 6, 3517–3524. (10.1534/g3.116.034678) 27591755 PMC5100850

[152] Rockman MV, Kruglyak L 2009 Recombinational landscape and population genomics of *Caenorhabditis elegans*. PLoS Genet. 5, e1000419 (10.1371/journal.pgen.1000419) 19283065 PMC2652117

[153] Kaur T, Rockman MV 2014 Crossover heterogeneity in the absence of hotspots in *Caenorhabditis elegans*. Genetics 196, 137–148. (10.1534/genetics.113.158857) 24172135 PMC3872180

[154] Smeds L, Mugal CF, Qvarnstrom A, Ellegren H 2016 High-resolution mapping of crossover and non-crossover recombination events by whole-genome re-sequencing of an avian pedigree. PLoS Genet. 12, e1006044 (10.1371/journal.pgen.1006044) 27219623 PMC4878770

[155] Singh ND, Stone EA, Aquadro CF, Clark AG 2013 Fine-scale heterogeneity in crossover rate in the *Garnet-Scalloped* region of the *Drosophila melanogaster* X chromosome. Genetics 194, 375–387. (10.1534/genetics.112.146746) 23410829 PMC3664848

[156] Adrian AB, Comeron JM 2013 The *Drosophila* early ovarian transcriptome provides insight to the molecular causes of recombination rate variation across genomes. BMC Genomics 14, 794 (10.1186/1471-2164-14-794) 24228734 PMC3840681

[157] Schultz J, Redfield H 1951 Interchromosomal effects on crossing over in *Drosophila*. Cold Spring Harb. Symp. Quant. Biol. 16, 175–197. (10.1101/SQB.1951.016.01.015) 14942738

[158] Lucchesi JC, Suzuki DT 1968 The interchromosomal control of recombination. Annu. Rev. Genet. 2, 53–86. (10.1146/annurev.ge.02.120168.000413)

[159] McVean GA, Myers SR, Hunt S, Deloukas P, Bentley DR, Donnelly P 2004 The fine-scale structure of recombination rate variation in the human genome. Science 304, 581–584. (10.1126/science.1092500) 15105499

[160] Auton A, McVean G 2007 Recombination rate estimation in the presence of hotspots. Genome Res. 17, 1219–1227. (10.1101/gr.6386707) 17623807 PMC1933511

[161] Wang Y, Rannala B 2008 Bayesian inference of fine-scale recombination rates using population genomic data. Phil. Trans. R. Soc. B 363, 3921–3930. (10.1098/rstb.2008.0172) 18852101 PMC2607416

[162] McVean G, Awadalla P, Fearnhead P 2002 A coalescent-based method for detecting and estimating recombination from gene sequences. Genetics 160, 1231–1241. 11901136 10.1093/genetics/160.3.1231 PMC1462015

[163] Hudson RR 2001 Two-locus sampling distributions and their application. Genetics 159, 1805–1817. 11779816 10.1093/genetics/159.4.1805 PMC1461925

[164] Fearnhead P, Donnelly P 2001 Estimating recombination rates from population genetic data. Genetics 159, 1299–1318. 11729171 10.1093/genetics/159.3.1299 PMC1461855

[165] Wang Y, Rannala B 2009 Population genomic inference of recombination rates and hotspots. Proc. Natl Acad. Sci. USA 106, 6215–6219. (10.1073/pnas.0900418106) 19342488 PMC2669376

[166] Clark AG, Wang X, Matise T 2010 Contrasting methods of quantifying fine structure of human recombination. Annu. Rev. Genomics Hum. Genet. 11, 45–64. (10.1146/annurev-genom-082908-150031) 20690817 PMC2980829

[167] Padhukasahasram B, Rannala B 2013 Meiotic gene-conversion rate and tract length variation in the human genome. Eur. J. Hum. Genet. (10.1038/ejhg.2013.30) 23443031

