# Supplementary material for: Background selection as null hypothesis in population genomics: insights and challenges from Drosophila studies
Source: Philos Trans R Soc Lond B Biol Sci. 2017 Nov 6;372(1736):20160471. doi: 10.1098/rstb.2016.0471 (PMC5698629; doi:10.1098/rstb.2016.0471)
Supplement: Population dynamics under varying intensities of BGS. [file rstb20160471supp1.pdf]

Supplementary Material for "Background selection as null hypothesis in population genomics: Insights and challenges from *Drosophila* studies"

Josep M. Comeron[1,2]

[1] Department of Biology, University of Iowa, IA 52242 USA, [2] Interdisciplinary Program in Genetics, University of Iowa, IA 52242 USA

I investigated population dynamics after a bottleneck and rapid recovery under varying intensities of background selection (BGS) using forward simulations (see figure 3 and text). Simulations were conducted using the program SLiM [1] and considered a panmictic population of 10,000 diploid individuals (*N*) at equilibrium (time 0), going through a severe bottleneck (0.22% of the initial population size) at time 0.1*N* and a rapid recovery to the initial *N* after 0.01*N* generations. The burn-in period was set to a minimum of 50*N* (500,000) generations to ensure stationary levels of polymorphism and linkage. Although shorter burn-in periods (~10*N*) may be adequate when BGS is strong (equivalent to a severely reduced N_e), longer periods are necessary to ensure equilibrium when following long sequences with high recombination rates.

All simulations followed a chromosome segment of 2 Mb that contains one representative *Drosophila* protein-encoding gene every 10 kb (solid lines) or every 50 kb (dashed lines). Each gene included a 1,000-bp 5′ UTR, a first 300-bp exon, a first intron of 1,000 bp, a second 600-bp exon, a short second intron of 200 bp, a third 600-bp exon, and a 300-bp 3′-UTR. The population mutation rate was set to be similar to that in *D. melanogaster* ($N \times u = 0.005$) and mutations were assigned to be either neutral or deleterious (see [2]). The proportion of deleterious mutations at the different genomic elements was set to 0.92 at first and second codon positions, 0.81 at UTRs, 0.56 at introns and 0.5 at intergenic sites [3, 4]; all third codon positions evolved neutrally. Following [5, 6], selection coefficients for deleterious mutations were assumed to follow a gamma distribution of fitness effects with mean $N \times s = -2{,}500$, a shape parameter *k* of 0.3, and a dominance coefficient *h* of 0.5.

Different degrees of BGS were generated by using different rates of total (sex-average) recombination realistic for *D. melanogaster*. Very strong BGS was accomplished with crossover (CO) rates (*c*) equivalent to 1×10^{-10}/bp/generation, strong BGS was accomplished with *c* equivalent to 1×10^{-9}/bp/generation and moderate BGS was accomplished with *c* equivalent to the genome-wide average ~ 1.2×10^{-8}/bp/generation. Rates of CO in the simulations, therefore, followed $N \times c$ of 0.0001 for very strong BGS (red line), 0.001 for strong BGS (green line) and 0.012 for moderate BGS (blue line), assuming that N_e for *D. melanogaster* is $\sim1\times10^{6}$. All simulations also included a non-crossover (NCO, or gene conversion) rate (*g*) of recombination equivalent to 4.8×10^{-8}/bp/generation for *D. melanogaster* (*N*

× g = 0.048) and a gene conversion tract length set to follow a geometric distribution with average 525 bp [7]. Within each recombination condition, two additional degrees of BGS were obtained by altering gene density (see above): 1 gene every 10 kb (solid lines, increased BGS) and 1 gene every 50 kb (dashed lines; reduced BGS).

I simulated a chromosome segment of 2 Mb to prevent underestimating the consequences of BGS and considered only the central 1 Mb region for evolutionary analyses to remove edge effects. Population parameters within this central 1 Mb region were estimated using a random sample of 20 chromosomes every 0.01N generations. Each data point in figure 3 depicts the mean of all non-overlapping 10-kb regions from 100 independent replicates. Relative levels of diversity at neutral sites are shown as π / π_0, where π_0 indicates neutral diversity at equilibrium (at time 0). Estimates of Tajima's D at neutral sites [8] are shown after normalizing by Dmin (D/Dmin) following [9]. Estimates of the fraction of adaptive amino acid substitutions α [10, 11] were obtained based on variation at first and second codon positions, using third codon positions as neutral and with values for fixed substitutions accumulating from time 0. Figure 3 shows estimates of α obtained using the DFE-alpha programs [12, 13] after jointly inferring the DFEs on amino acid mutations and demography under a two-epoch model.

References

1. Messer, P.W. 2013 SLiM: simulating evolution with selection and linkage. *Genetics* **194**, 1037-1039. (doi:10.1534/genetics.113.152181).
2. Comeron, J.M. 2014 Background selection as baseline for nucleotide variation across the *Drosophila* genome. *PLoS Genet* **10**, e1004434. (doi:10.1371/journal.pgen.1004434).
3. Casillas, S., Barbadilla, A. & Bergman, C.M. 2007 Purifying selection maintains highly conserved noncoding sequences in *Drosophila*. *Mol Biol Evol* **24**, 2222-2234. (doi:10.1093/molbev/msm150).
4. Sella, G., Petrov, D.A., Przeworski, M. & Andolfatto, P. 2009 Pervasive natural selection in the *Drosophila* genome? *PLoS Genet* **5**, e1000495. (doi:10.1371/journal.pgen.1000495).
5. Charlesworth, B. 2012 The role of background selection in shaping patterns of molecular evolution and variation: evidence from variability on the *Drosophila* X chromosome. *Genetics* **191**, 233-246. (doi:10.1534/genetics.111.138073).
6. Haddrill, P.R., Loewe, L. & Charlesworth, B. 2010 Estimating the parameters of selection on nonsynonymous mutations in *Drosophila pseudoobscura* and *D. miranda*. *Genetics* **185**, 1381-1396. (doi:10.1534/genetics.110.117614).

7. Comeron, J.M., Ratnappan, R. & Bailin, S. 2012 The many landscapes of recombination in *Drosophila melanogaster*. *PLoS Genet* **8**, e1002905. (doi:10.1371/journal.pgen.1002905).

8. Tajima, F. 1989 Statistical method for testing the neutral mutation hypothesis by DNA polymorphism. *Genetics* **123**, 585-595.

9. Schaeffer, S.W. 2002 Molecular population genetics of sequence length diversity in the *Adh* region of *Drosophila pseudoobscura*. *Genet Res* **80**, 163-175.

10. Fay, J.C., Wyckoff, G.J. & Wu, C.I. 2001 Positive and negative selection on the human genome. *Genetics* **158**, 1227-1234.

11. Smith, N.G. & Eyre-Walker, A. 2002 Adaptive protein evolution in *Drosophila*. *Nature* **415**, 1022-1024.

12. Keightley, P.D. & Eyre-Walker, A. 2007 Joint inference of the distribution of fitness effects of deleterious mutations and population demography based on nucleotide polymorphism frequencies. *Genetics* **177**, 2251-2261. (doi:10.1534/genetics.107.080663).

13. Eyre-Walker, A. & Keightley, P.D. 2009 Estimating the rate of adaptive molecular evolution in the presence of slightly deleterious mutations and population size change. *Mol Biol Evol* **26**, 2097-2108. (doi:10.1093/molbev/msp119).
